# Effects of mechanical stress on chondrocyte phenotype and chondrocyte extracellular matrix expression

**DOI:** 10.1038/srep37268

**Published:** 2016-11-17

**Authors:** Qiang Liu, Xiaoqing Hu, Xin Zhang, Xiaoning Duan, Peng Yang, Fengyuan Zhao, Yingfang Ao

**Affiliations:** 1 Institute of Sports Medicine, Beijing Key Laboratory of Sports Injuries, Peking University Third Hospital, 49 North Garden Road, Haidian District, Beijing 100191, P. R. China

## Abstract

Mechanical factors play a key role in regulating the development of cartilage degradation in osteoarthritis. This study aimed to identify the influence of mechanical stress in cartilage and chondrocytes. To explore the effects of mechanical stress on cartilage morphology, we observed cartilages in different regions by histological and microscopic examination. Nanoindentation was performed to assess cartilage biomechanics. To investigate the effects of mechanical stress on chondrocytes, cyclic tensile strain (CTS, 0.5 Hz, 10%) was applied to monolayer cultures of human articular chondrocytes by using Flexcell-5000. We quantified the mechanical properties of chondrocytes by atomic force microscopy. Chondrocytes were stained with Toluidine blue and Alcian blue after exposure to CTS. The expression of extracellular matrix (ECM) molecules was detected by qPCR and immunofluorescence analyses in chondrocytes after CTS. Our results demonstrated distinct morphologies and mechanical properties in different cartilage regions. In conclusion, mechanical stress can affect the chondrocyte phenotype, thereby altering the expression of chondrocyte ECM.

Osteoarthritis (OA) is a degenerative joint disease characterized by articular cartilage degradation; such degradation is the leading cause of physical disability^1^. OA is considered a heterogeneous group of disorders with various pathogenic factors, which result in similar patterns of cartilage degeneration^2^. Cartilage cellularity in OA is reduced by chondrocyte death; chondrocytes are stimulated by cytokines and growth factors to a catabolic and abnormal differentiation leading to extracellular matrix (ECM) degradation^3^,^4^. The degradation of ECM is complicated because it involves genetic, developmental, biochemical, and biomechanical factors. A single joint with the same genetic, developmental, and biochemical background exhibits varying degrees of cartilage injury; generally, the medial compartment of the articular cartilage is the most susceptible to degeneration, whereas the lateral compartment remains relatively unaffected^5^,^6^. The strongest reason is the mechanical factor; chondrocytes residing in damaged regions are susceptible to mechanical stress, and the tensile properties of the damaged cartilage are lost from the destruction of the collagen network^7^,^8^.

As mechanosensitive cells, chondrocytes perceive and respond to mechanical stress throughout life. Chondrocytes synthesize the extracellular matrix and their integrity; in addition, chondrocytes depend upon intracellular signals generated in response to biomechanical stress^9^,^10^,^11^. Chondrocytes maintain a functional balance between degradation and repair by producing various enzymes, cytokines, and matrix-associated proteins. A loss of this functional balance is associated with changes in their phenotypic characteristics^12^. Phenotypically, articular chondrocytes are characterized by their ability to synthesize a specific matrix consisting of type II collagen and aggrecan; this matrix allows them to withstand changes in their mechanical environment^13^,^14^,^15^.

In the present study, we investigated the morphologies and mechanical properties in different cartilage regions. We examined the mechanical stress in chondrocytes by using Flexcell-5000 and explored the effects of CTS on the chondrocyte phenotype. The chondrocyte ECM expression was detected by qPCR and immunofluorescence analyses.

## Results

### Histologic examination of cartilage in different regions

**Figure 1:**
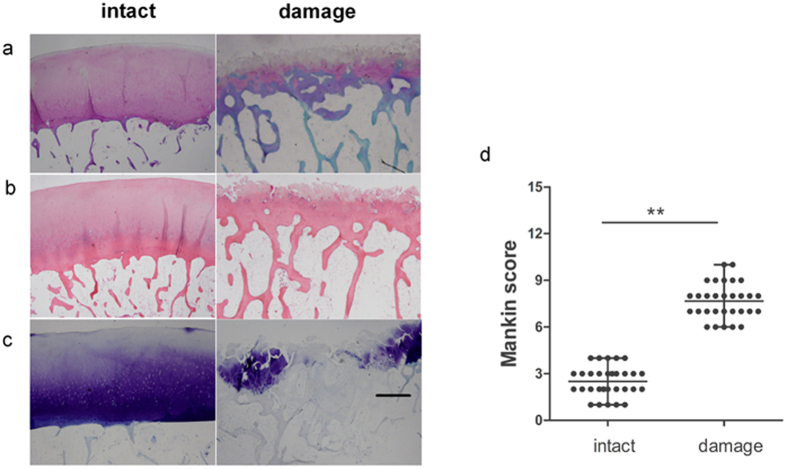
Histologic examination of cartilage in different regions. (**a**) Hematoxylin and eosin staining of cartilage in different regions. (**b**) Toluidine blue staining of cartilage in different regions. (**c**) Sanfranin O–Fast green staining of cartilage in different regions. Scale bars = 500 μm. (**d**) Mankin scores of intact vs. damaged cartilage. Presented values are mean ± SEM, *P < 0.05.

In order to observe the morphologies of cartilage in intact and damage region, we examined the cartilage by histologically. The sections were stained with safranin-O, hematoxylin and eosin, as well as Toluidine blue. The cartilage samples with smooth surface, intact hyaline cartilage architecture, and no loss of proteoglycan staining were defined as intact regions of the cartilage with OA. By contrast, cartilage samples with fibrillation, clefts, several cell clusters, and extensive loss of proteoglycan staining were defined as damaged regions of the cartilage with OA (Fig. 1a–c). The histologic score of the damaged region (mean ± SD, 8 ± 1) was significantly higher than that of the intact region (3 ± 1) (Fig. 1d).

### Observation of cartilage in different regions with TEM and SEM

**Figure 2:**
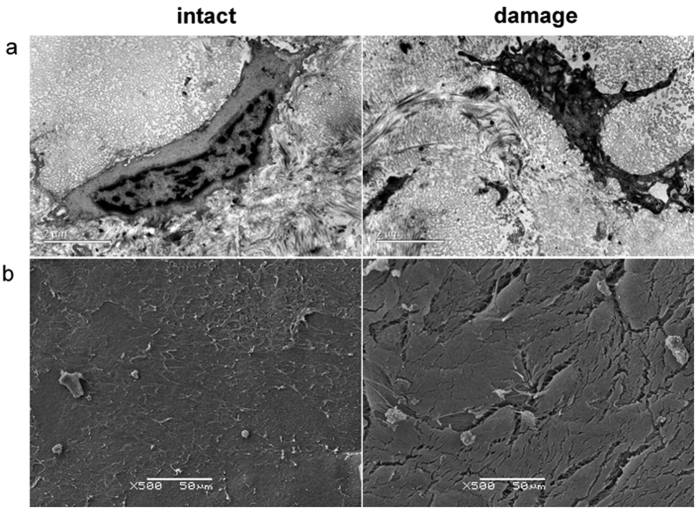
Observation of cartilage with TEM and SEM. (**a**) TEM imaging of the cartilage from intact and damaged regions in OA. Scale bars = 2 μm. (**b**) SEM imaging of the surface of the intact and damaged regions in OA. Scale bars = 50 μm.

In order to observe the microstructure of the cartilage in the intact and damaged regions, we examined the cartilage by TEM and SEM. In the intact regions, the size of the chondrocyte was relatively normal, and the cell outline was intact and clear; the cellular projections were visible, and the collagen texture was slightly indistinct. In the damaged regions, the amount of finely granular matter increased; similarly, the projections on the chondrocyte surface increased. Meanwhile, the chondrocyte nuclei were deformed, with uneven chromatin distribution and visible fat droplets. The matrix fibers became more cluttered, and the cell outline was unclear or even missing (Fig. 2a). SEM images showed that the articular cartilage in the intact region indicated a relatively normal organization with an undulated course on the surface; this cartilage also showed a superficial cobblestone-like appearance. SEM images showed changes in the structural organization of the damaged region; it exhibited a rough surface with irregular grooves, and the matrix fibers were arranged more loosely; furthermore, the connections were blocked (Fig. 2b).

### Assessment of cartilage biomechanics with nanoindentation

**Figure 3:**
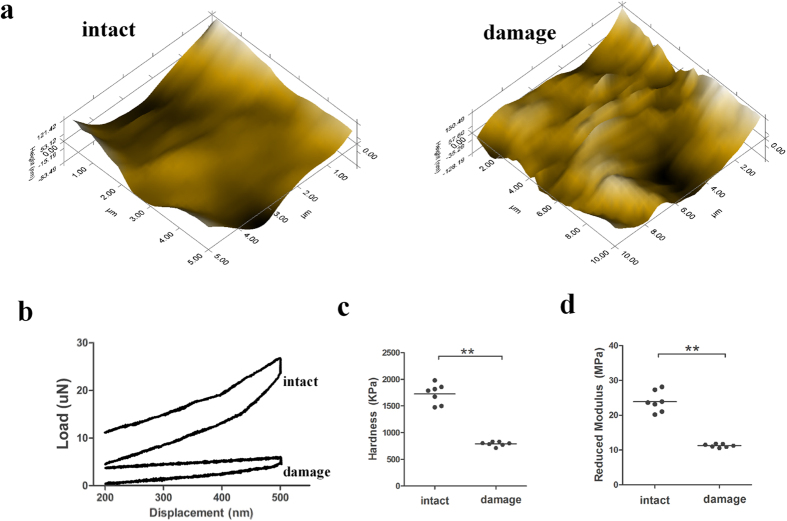
Assessment of cartilage biomechanics by nanoindentation. (**a**) Microscopic geomorphology of the different regions was acquired during nanoindentation. (**b**) Typical load–displacement curves of two groups were recorded within a test range of 500 nm. (**c**) The biomechanical properties of hardness were calculated with the biomechanical curves. (**d**) The biomechanical properties of the reduced modulus were calculated with the biomechanical curves (*n* = 5, *p < 0.05, **p < 0.01).

In order to detect biomechanics of the intact and damage cartilage, we performed nano-indentation to assess cartilage biomechanics. Micro-scanning images showed that the articular surface in the damaged region was scraggier and rougher than the intact region, which is consistent with SEM and TEM analyses (Fig. 3a). By using load–displacement curves, we quantified the biomechanical properties of the two regions (Fig. 3b). The cartilage in the intact region was harder than that in the damaged region (Fig. 3c). Moreover, the reduced modulus value of the intact cartilage was significantly higher than that of the damaged cartilage (Fig. 3d). Therefore, nanoindentation testing indicated that the different regions of cartilage exhibited distinct biomechanical properties.

### Chondrocytes were stimulated by CTS and observed via TEM and AFM

**Figure 4:**
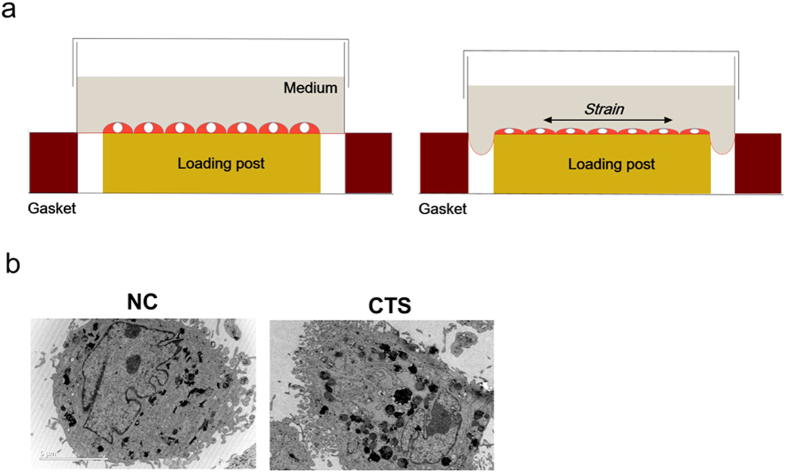
Chondrocytes were stimulated by CTS and observed by TEM. (**a**) Schematic diagram of the FX-5000 Flexcell system. (**b**) TEM imaging of chondrocytes stimulated with CTS and control. Scale bars = 5 μm.

In order to mimic the cartilage stimulated with stress *in vivo*, we established a model of chondrocytes exposed to CTS *in vitro* and observed chondrocytes by SEM. To investigate the biomechanics of chondrocytes, cells were exposed to pre-optimized magnitudes of CTS by using Flexcell-5000 (Fig. 4a). Chondrocytes were exposed to CTS with a magnitude of 10% (0.5 Hz) for 24 h throughout the study. According to TEM analysis, unstimulated chondrocytes showed a euchromatic nucleus, a cytoplasm with an abundant rough endoplasmic reticulum, and several cytoplasmic processes. However, chondrocytes exposed to mechanical stress showed a reduced size of the nucleus with partially condensed chromatin; the cytoplasm contained the swollen rough endoplasmic reticulum and numerous vesicles (Fig. 4b).

**Figure 5:**
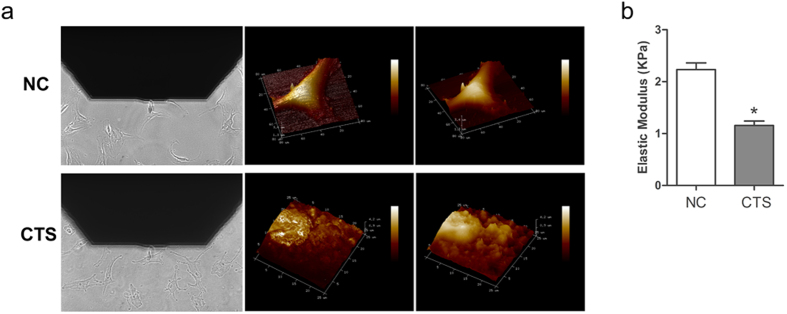
Chondrocytes were observed by AFM. (**a**) Image of the alignment of the AFM tip/bead over a single chondrocyte and the 3d-height representation of the scanned area in chondrocytes with CTS and control. (**b**) Statistical quantitative comparison of average elastic modulus for chondrocytes with CTS and control. For each type of sample, three different areas were considered, and calculations were conducted via bearing analysis on Peak Force Tapping images. Presented results are mean ± SEM of at least three independent experiments (*p < 0.05, **p < 0.01).

In order to determine the biomechanical property of chondrocytes exposed to CTS, we performed atomic force microscopy (AFM) and detected the elastic modulus. Topography images showed that the surface of chondrocytes with CTS was rough, compared with the unstimulated chondrocytes with smooth surface (Fig. 5a and b). The results indicated that the elastic modulus decreased in chondrocytes stimulated with CTS compared with the unstimulated chondrocytes (Fig. 5c).

### Effects of biomechanical stress on chondrocyte ECM expression

**Figure 6:**
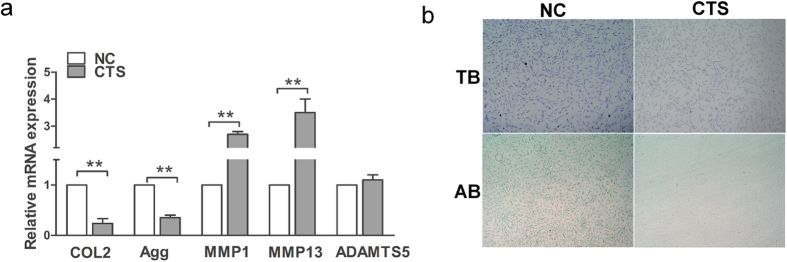
Effects of biomechanical stress on chondrocyte ECM expression. (**a**) The expression of COL2A1, aggrecan, MMP1, MMP13, and ADAMTS5 was analyzed by qPCR in chondrocytes with CTS and control. The results are expressed as mean ± SEM of at least three independent experiments (*P < 0.05, **p < 0.01). (**b**) Toluidine blue staining and Alcian blue staining for chondrocytes stimulated with CTS and control.

**Figure 7:**
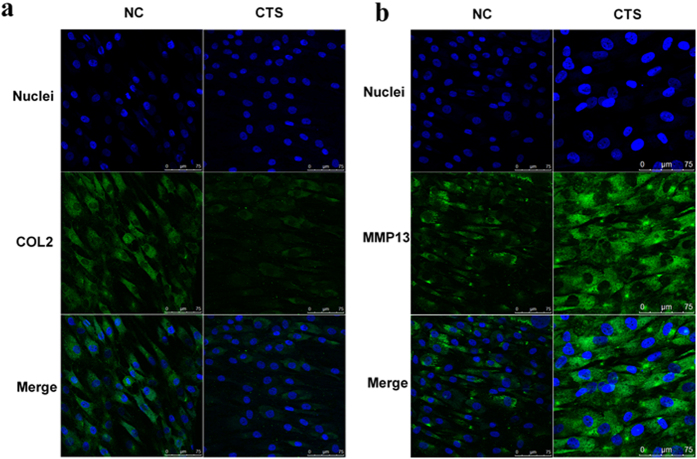
Immunofluorescence analysis of COL2 and MMP13 expression in chondrocytes. (**a**,**b**) Immunofluorescence staining for COL2 and MMP13 in chondrocyte with CTS and control. Specific antibodies against type II collagen and matrix metalloproteinase 13 were used, with fluorescein isothiocyanate and Hoechst 33342 staining. Bars = 75 μm; original magnification ×40.

We presume that biomechanical stress could induce the matrix degradation and decrease the matrix synthesis. As mentioned above, chondrocytes were exposed to pre-optimized magnitudes of CTS; the expression levels of COL2A1 and aggrecan were significantly downregulated, whereas the expression levels of MMP1 and MMP13 were significantly upregulated, and ADAMTS5 expression was essentially unaffected by CTS (Fig. 6a). Furthermore, chondrocytes were stained with Toluidine blue and Alcian blue. The results showed that the chondrocytes stimulated with CTS included fewer ECM components than unstimulated cells (Fig. 6b). The ECM-related proteins COL II and MMP13 were also evaluated by immunofluorescence staining. The results showed that the expression of COL II decreased, whereas the expression of MMP13 increased in chondrocytes after CTS (Fig. 7).

## Discussion

OA is a multifactorial disease characterized by progressive inflammation, pain, and cartilage destruction in load-bearing surfaces of knee joints^16^. Among various pathogenic factors, biomechanical factor is critical for cartilage development, homeostasis, and functionality^17^. Aberrant mechanical stimulation results in a physiological imbalance between mechanical stress on the joint and the joint’s ability to withstand such stress. Chondrocytes are directly exposed to compression force during cartilage loading; thus, collagen and other matrix components of the ECM interaction linked to the chondrocytes will likely stretch the cells during cartilage compression^18^. During normal movements *in vivo*, articular chondrocytes are exposed to compression loads of 15%, leading to 5% tensile strain in chondrocytes^19^,^20^. Previous studies have confirmed that chondrocytes perceive mechanical stress and respond to it in a magnitude-dependent manner. The mechanical stressors of low magnitude (2% to 8% CTS) were not perceived as inflammatory signals, whereas CTS of high magnitudes was associated with proinflammatory gene expression, which markedly upregulated the matrix degradation and decreased the matrix synthesis^20^,^21^,^22^,^23^. Our study applied CTS with 10% elongation for 24 h in mechanical stress.

A previous study found that the elastic modulus of chondrocytes in all age groups decreased with increased indentation (15 nm to 2000 nm). The elastic modulus of adult chondrocytes was significantly greater than that of neonatal cells at indentations greater than 500 nm. Furthermore, the intrinsic viscosity was lower in geriatric chondrocytes than in neonatal ones^24^. In addition, the stiffness of chondrocytes obtained from older human individuals (>55 years old) was greater than that of chondrocytes obtained from younger human individuals (18–35 years old)^25^. The changes in the elastic and viscoelastic mechanical properties of the cells may influence the synthetic activity of the chondrocytes, which are known to respond to their mechanical environment^26^,^27^,^28^. Our study is the first to investigate the mechanics of chondrocytes stimulated by CTS; the elastic moduli decreased in chondrocytes stimulated with CTS compared with the unstimulated chondrocytes.

Many biological processes were involved in mechanical stress, which included but were not limited to the recruitment of adapter proteins and the activation of protein kinases, such as extracellular-signal-related kinase (ERK), c-Jun N-terminal kinase (JNK), and p38. Consequently, these kinases activate certain transcription factors, thereby resulting in changes in gene expression and metabolism^29^. Mechanical stress has also been reported in chondrocytes, but the present study focused on the effects of biomechanical stress on the cell phenotype and ECM expression in human chondrocytes. The mechanisms by which mechanical stress regulates these changes are not fully understood but are likely interconnected because the chondrocyte mechanical properties, phenotype, and ECM had all been linked to the cytoskeleton structure^30^,^31^. Many studies have proposed a relationship between the cytoskeleton and biological phenotype from healthy differentiated cells to cancer cells^32^,^33^,^34^. These findings showed that both the elastic and viscoelastic properties of chondrocytes can be significantly altered by treatment with agents that disrupted or enhanced the structure of F-actin microfilaments^31^,^35^. Thus, we presumed that mechanical stress can induce ECM expression by regulating the cytoskeleton arrangement and expression. Collectively, our results demonstrated that different cartilage regions indicated distinct morphologies and mechanical properties. Furthermore, mechanical stress can affect the chondrocyte phenotype and alter the expression of chondrocyte ECM.

## Materials and Methods

### Patients and specimens

OA cartilage was isolated from the knee joints of 30 patients undergoing total knee arthroplasty. The tissues were processed for histological examination. The informed consent was obtained from all subjects. This study was approved by the Human Ethics Committee of Peking University Third Hospital (China). The methods were performed in accordance with the approved guidelines.

### Histological examination

Cartilage specimens were dehydrated in graded alcohols and xylene, embedded in paraffin, and cut serially into 5 mm sagittal sections. The sections were stained with Toluidine blue, safranin-O, as well as hematoxylin and eosin as routine protocol. Changes were graded based on the modified Mankin scale^36^. A score of <4 points was considered intact cartilage, and a score of >6 represented damaged cartilage^37^.

### TEM/SEM

Cartilage specimens were subjected to fixation, gradient dehydration, and embedment. The specimens were sliced into ultra-thin sections, mounted on copper grids, and then stained with uranyl acetate and lead citrate^38^. The cells were grown on pronectin-coated Bioflex six-well culture plates and they were collected by scraping from the plates before fixing in cold Karnovsky fixative for TEM analysis. Afterward, the specimens were observed by TEM, with a Philips EM 208 TEM (Philips Scientifics, Eindhoven, Netherlands). For SEM, the specimens were dehydrated, mounted on aluminum stubs, coated with gold, and then examined using high-resolution SEM S-2500 (Hitachi Ltd., Tokyo, Japan).

### Nanoindentation/AFM

Biomechanical analysis of cartilage tissues was performed using nanoindentation. Samples were isolated from different cartilage regions. All indentations were performed using the TriboIndenter (Hysitron Inc., Minneapolis, MN, USA) with a 100-mm radius curvature conospherical diamond probe tip. A trapezoidal load function was applied to each indent site with loading (10 s), holding (2 s), and unloading (10 s). The microscopic geomorphology of the indentation zones was captured using a micro-scanning apparatus^39^. The mechanical properties of the chondrocytes were measured using a Bioscope Resolve AFM (Bruker AXS Corporation, Santa Barbara, CA), following previously established techniques^40^. The light microscope used was DMI6000 (Leica, Wetzlar, Germany). For elastic modulus measurements, chondrocytes were maintained in the medium at room temperature. All presented data were acquired on Bioscope Catalyst^TM^ (Veeco Instruments, Santa Barbara CA, USA).

### Culture of primary chondrocytes and exposure to mechanical stress

Donor chondrocytes were isolated as previously described^41^. The chondrocytes were at passage 2 and fibroblasts were used as control. We detect specific markers in both cells by immunofluorescence analysis. The results showed that the expression of Sox9 is high in our chondrocytes, while low in fibroblasts (Supplementary Figure 1A). Meanwhile, expression of S100A4 is low in our chondrocytes, while high in fibroblasts (Supplementary Figure 1B). Chondrocytes (5 × 10^5^/well) were grown on pronectin-coated Bioflex six-well culture plates (Flexcell International, Hillsborough, NC) to 80% confluence. Cyclic tensile strain (CTS) experiments were performed using the FX-5000 Flexcell system (Flexcell International, McKeesport, PA). CTS was enforced at 5%, 10%, and 15% elongation (0.5 Hz) for 24 h^42^,^43^.

### Quantitative real-time PCR

Total RNA was isolated from cartilage tissues or chondrocytes by using TRIzol reagent. Isolated RNA was reverse transcribed with the use of a kit (Promega), and qPCR was performed with the Mx3005 real-time PCR system (Agilent). The expression of mRNAs relative to GAPDH was determined using the 2-ΔΔCT method^44^. The following primers were used in this study:

COL2 forward: 5′-TGGACGATCACGAAACC-3′, reverse: 5′-GCTGCGGATGCTCTCAATCT-3′; aggrecan forward: 5′-ACTCTGGGTTTTCGTGACTCT-3′, reverse: 5′-ACACTCAGCGAGTTGTCATGG-3′; MMP13 forward: 5′-ACTGAGAGGCTCCGAGAAATG-3′, reverse: 5′-GAACCCCGCATCTTGGCTT-3′; ADAMTS5 forward: 5′-GAACATCGACCAACTCTACTCCG-3′, reverse: 5′-CAATGCCCACCGAACCATCT-3′.

### Immunofluorescence analysis

Cells were rinsed in PBS and fixed with 4% paraformaldehyde for 15 min at room temperature. Goat serum was used to block nonspecific binding sites. Cultured cells were incubated with anti-COL2A1 (1:200 dilution), anti-SOX9 (1:200 dilution), anti-S100A4 (1:200 dilution) and anti MMP13 (1:200 dilution) at 4 °C overnight. Cells were incubated for 1 h with goat anti-rabbit IgG (1:100 dilution). Cells were observed under a confocal microscope (FV 1000 Olympus IX-81, Olympus, Tokyo, Japan)^41^. Immunofluorescence images were analyzed using Image-Pro Plus 6.0 software (Media Cybernetics, Silver Spring, MD).

### Statistics

Statistically significant differences from multiple groups were calculated using analysis of variance. Results from the Mankin Score were evaluated using non-parametric tests (chi-square test). Results from the same group were evaluated using *t*-tests. The results are reported as mean ± SEM. P values less than 0.05 were considered statistically significant. All experiments were performed and analyzed in triplicate. Data analysis was conducted using SPSS software.

## Additional Information

**How to cite this article**: Liu, Q. *et al.* Effects of mechanical stress on chondrocyte phenotype and chondrocyte extracellular matrix expression. *Sci. Rep.* **6**, 37268; doi: 10.1038/srep37268 (2016).

**Publisher’s note:** Springer Nature remains neutral with regard to jurisdictional claims in published maps and institutional affiliations.

## Supplementary Material

Supplementary Information
