# Supplementary material for: Effects of mechanical stress on chondrocyte phenotype and chondrocyte extracellular matrix expression
Source: Sci Rep. 2016 Nov 17;6:37268. doi: 10.1038/srep37268 (PMC5112533; doi:10.1038/srep37268)
Supplement: Supplementary Information [file srep37268-s1.pdf]

Effects of mechanical stress on chondrocyte phenotype and chondrocyte extracellular matrix expression

Qiang Liu[1], Xiaoqing Hu[1], Xin Zhang[1], Xiaoning Duan[1], Peng Yang[1], Fengyuan Zhao[1], Yingfang Ao[1*]

1 Institute of Sports Medicine, Beijing Key Laboratory of Sports Injuries, Peking University Third Hospital, 49 North Garden Road, Haidian District, Beijing 100191, P. R. China.

Correspondence authors' Address:

*Institute of Sports Medicine, Peking University Third Hospital, 49 North Garden Road, Haidian District, Beijing 100191, P. R. China. (Yingfang Ao, MD)

Fax number: 86-10-62010440; Tel. 86-10-82267390; Email: yingfang.ao @gmail.com;

Supplementary figure 1.

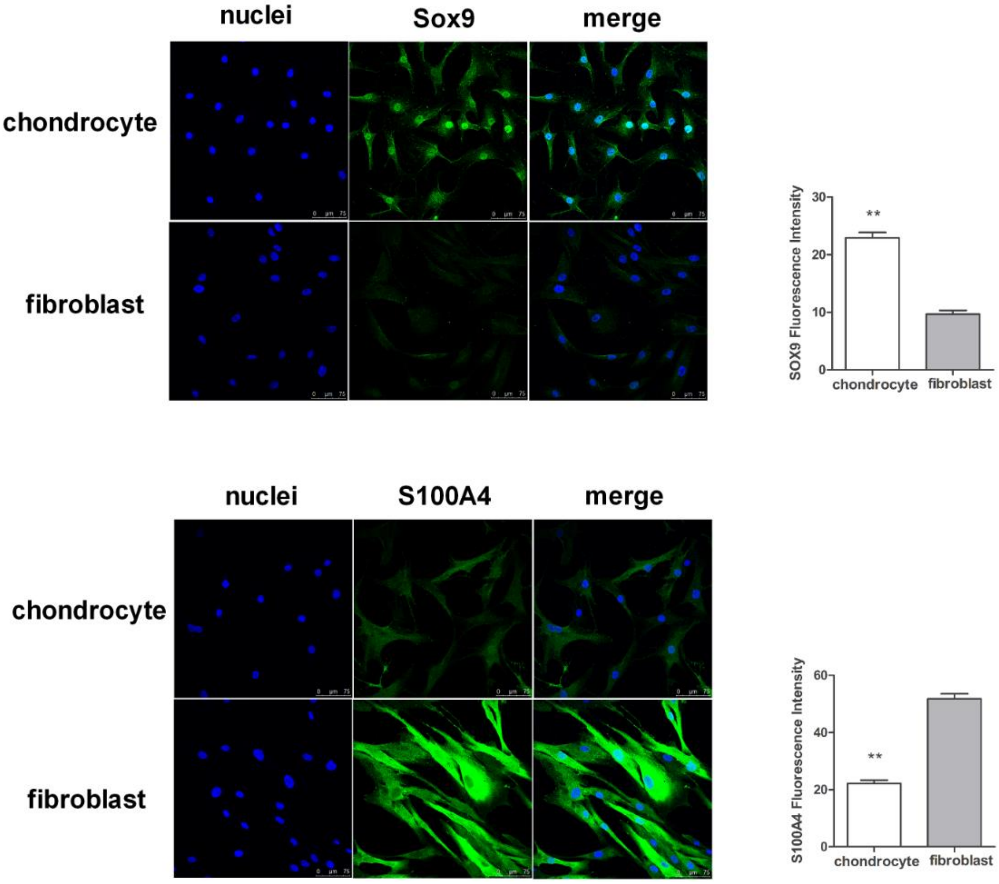

Supplementary figure 1. (A, B) Immunofluorescence analysis of SOX9 and S100A4 expression in chondrocytes and fibroblasts. Specific antibodies against SOX9 and S100A4 were used, with fluorescein isothiocyanate and Hoechst 33342 staining.
